# Direct and indirect effects of coaching leadership style on athlete engagement

**DOI:** 10.3389/fpsyg.2025.1531049

**Published:** 2025-09-17

**Authors:** Jingjing Li, Suxuan Xing

**Affiliations:** ^1^College of Physical Education (Gymnastics Academy), Chengdu Sport University, Eastern New District, Chengdu, China; ^2^College of Sport Training, Chengdu Sport University, Eastern New District, Chengdu, China

**Keywords:** coaching leadership style, athletic engagement, perceived social support, achievement goal orientation, chain mediating effects

## Abstract

**Introduction:**

This study examines how coaching leadership styles influence athlete engagement through the sequential mediating mechanisms of perceived social support and achievement goal orientation.

**Methods:**

Using validated psychological scales, the research explores the interplay between democratic and authoritarian leadership approaches, athletes’ perceptions of social support, and their task- or ego-oriented goal pursuits.

**Results:**

The findings reveal that democratic leadership significantly enhances athlete engagement by fostering a supportive environment that strengthens perceived social support, which subsequently promotes task-oriented goals. This dual pathway highlights the critical role of social support and mastery-focused motivation in cultivating athlete engagement. In contrast, authoritarian leadership does not demonstrate meaningful associations with engagement or its mediators.

**Discussion:**

The results underscore the importance of democratic leadership in creating a psychologically empowering climate, offering actionable insights for coach development programs to emphasize autonomy-supportive and collaborative strategies. Theoretically, the study bridges Self-Determination Theory and Achievement Goal Theory by demonstrating how socially embedded support systems and task-oriented goals synergistically contribute to optimizing athlete performance and well-being.

## Introduction

The coach-athlete relationship constitutes a dynamic, symbiotic microsystem (Li, 2021) in which leadership styles simultaneously shape the immediate training climate and, via person-behavior-environment transactions (Bandura, 1978), subtly govern how fully athletes invest themselves and how effectively they perform. Self-Determination Theory maintains that satisfaction of the basic psychological needs for autonomy, competence and relatedness is essential for sustaining intrinsic motivation (Deci and Ryan, 1985; Ryan and Deci, 2000), whereas situational leadership perspectives remind us that athletes’ technical and psychological states are in constant flux (Chelladurai and Saleh, 1980). Coaches must therefore continuously recalibrate their behaviors in response to these fluctuations (Fiedler, 1967; House, 1971; Hersey and Blanchard, 1982; Cahyono et al., 2023) so that optimal levels of support and challenge are consistently supplied (Mageau and Vallerand, 2003).

Within this process, leadership style first colours athletes’ subjective appraisal of available support. When coaches adopt a democratic orientation—characterized by open communication, emotional concern and structured guidance rather than unilateral command (González-García et al., 2021; Jowett et al., 2016; Podlog et al., 2015)—athletes experience greater respect and understanding (Gabana et al., 2022). Consequently, support originating from coaches, teammates and family is internalized as psychological safety (Zimet et al., 1990; Poucher et al., 2018), effectively buffering stress reactions during training and competition (Katagami and Tsuchiya, 2016; Liu et al., 2023). This heightened perceived social support not only elevates engagement directly (Hodge et al., 2009; Miao et al., 2022) but also strengthens self-efficacy, thereby guiding athletes toward a task-oriented achievement goal that centres on self-referenced skill improvement rather than normative superiority (Wentzel, 1994; Hernandez et al., 2016; Martínez-López et al., 2024). Task orientation, in turn, is associated with robust self-efficacy and positive achievement emotions (Bandura and Kavussanu, 2018; Miller et al., 2021), which feed back to consolidate intrinsic motivation and psychological well-being (Jakobsen, 2021; Wekesser et al., 2021), producing a cascading “leadership → support → goal → engagement” sequence.

Conversely, an over-reliance on authoritarian leadership may instil discipline in the short term (Bass and Riggio, 2006) yet simultaneously undercut athletes’ sense of autonomy (Vallerand, 1997), diminish favorable appraisals of support, and precipitate ego-oriented social comparisons and performance anxiety (Martins et al., 2017; Elbe et al., 2023), ultimately undermining sustained engagement and health (Jin et al., 2022).

Integrating Self-Determination Theory with achievement-goal perspectives, the present research therefore examines the chain-mediated pathway through which coaching leadership styles influence athlete engagement via perceived social support and achievement goal orientation. The findings are expected to provide both theoretical depth and actionable evidence for the precision-tuning of coaching behaviors in high-performance sport settings (Reynders et al., 2019; Borghi et al., 2017).

In summary, we developed hypotheses H1a, H1b, H2a, H2b, H3a, H3b, H4a, H4b.

H1a: Coaches’ democratic leadership style has a significant positive effect on athlete engagement.

H1b: Coaches’ authoritarian leadership style has a significant negative effect on athlete engagement.

H2a: Perceived social support mediates the relationship between coaches’ democratic leadership style and athlete engagement.

H2b: Perceived social support mediates the relationship between coaches’ authoritarian leadership style and athlete engagement.

H3a: Athlete task orientation influences the relationship between democratic leadership styles and athlete engagement.

H3b: Athlete ego orientation influences the relationship between authoritarian leadership styles and athlete engagement.

H4a: perceived social support and athlete task orientation act as chain mediators between democratic leadership styles and athlete engagement.

H4b: perceived social support and athlete ego orientation act as chain mediators between authoritarian leadership styles and athlete engagement.

### Current research

**Figure 1:**
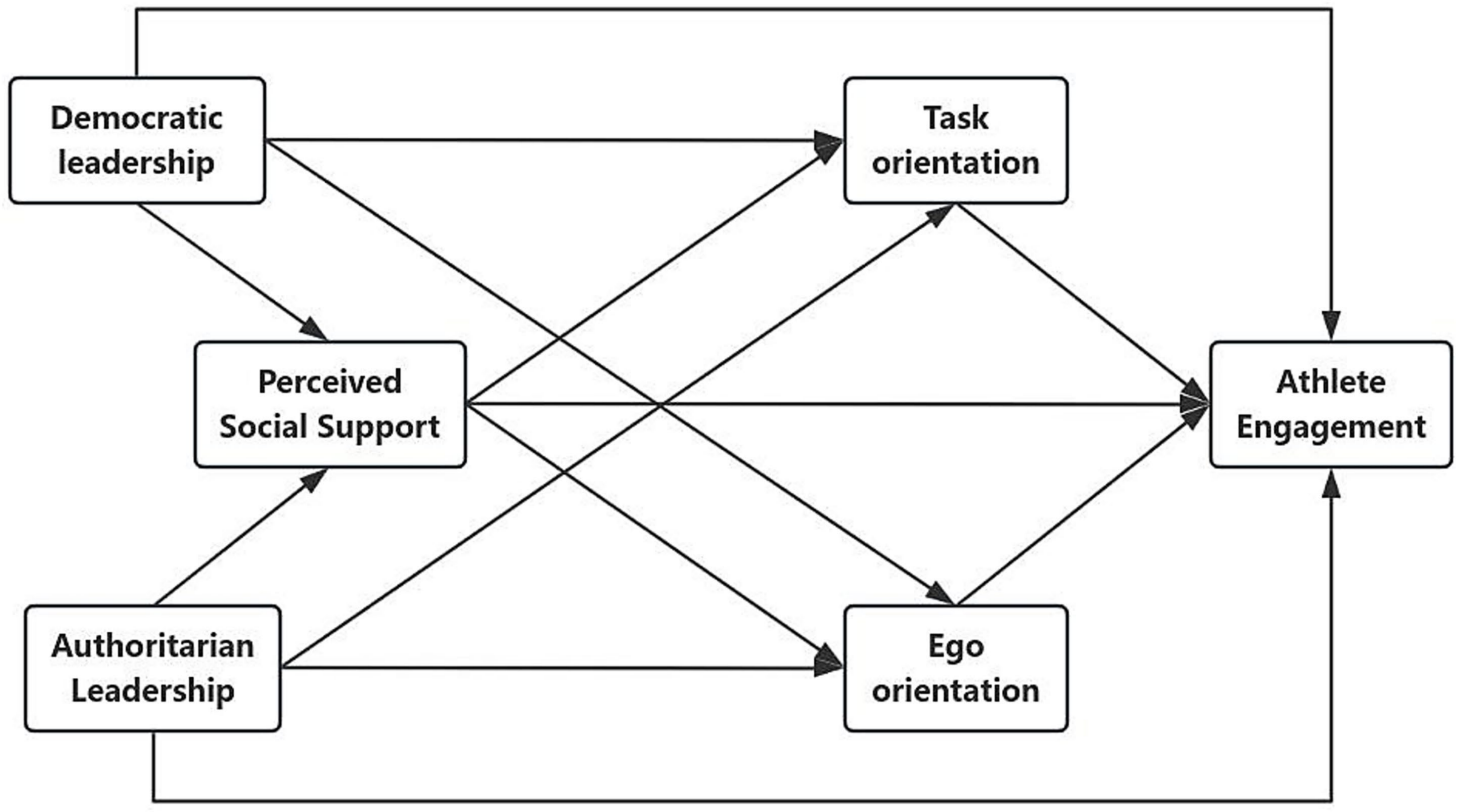
The proposed multiple mediation model.

The present study constructed a chain mediation model in order to deeply explore the mechanism of the influence of coaches’ leadership styles on athlete engagement and the mediating roles of perceived social support and goal-achievement orientation between them, aiming to provide new ideas for the effect enhancement and effective intervention strategies of athlete engagement (see Figure 1).

## Materials and methods

### Data collection methods

The study received ethical approval from Chengdu Sport University’s Institutional Review Board (Approval No.: 2024–90). Used G*Power 3.1.9.7 for a multiple-regression model with five predictors, *α* = 0.05, powe*r* = 0.80, and a medium effect size f^2^ = 0.15; the required *N* = 119. To offset potential attrition and allow subgroup analyses, we doubled this number to 240 and ultimately collected 290 valid responses. From May 13 to June 20, 2024, the research team recruited 300 athletes through on-site visits and email contacts across four Chinese provinces: Sichuan, Shandong, Guangdong, and Heilongjiang. Interested athletes received detailed information about the study’s purpose, requirements, and expectations, along with an informed consent form. Participants who signed the consent form completed a multi-part online questionnaire via the “Questionnaire Star” platform. A total of 290 valid responses were collected, representing both individual and team sports, including field hockey (*n* = 38, 13.1%), sparring (*n* = 30, 10.3%), basketball (*n* = 24, 8.3%), and artistic gymnastics (*n* = 23, 7.9%). Athletics (*n* = 70, 24.1%), Swimming (*n* = 55, 19.0%), Badminton (*n* = 30, 10.3%), Shooting (*n* = 20, 6.9%).

Participant demographic characteristics are summarized in Table 1. The sample included 147 female athletes (50.7%) and 143 male athletes (49.3%), aged 13–34 years (mean age *M* = 18.73, SD = ±3.72). Average training experience was 6.87 years (SD = ±4.45). Competitive levels comprised: “National Master Athlete” (*n* = 88, 30.3%), “National Level Athlete” (*n* = 69, 23.8%), “National Secondary Level Athlete” (*n* = 49, 16.9%), and “Developmental Athletes” (*n* = 84, 29.0%).

**Table 1:** Demographic characteristics of participants (*N* = 290).

Variable	Category	Count (Percentage)
Gender	Female	147 (50.7%)
	Male	143 (49.3%)
Age	13–17 years	124 (42.8%)
	18–25 years	136 (46.9%)
	≥26 years	30 (10.3%)
Sport type	Individual	142 (49.0%)
	Team	148 (51.0%)
Competitive level	National Master Athlete	88 (30.3%)
	National Level Athlete	69 (23.8%)
	National Secondary Level Athlete	49 (16.9%)
	Developmental Athletes	84 (29.0%)
Training experience	≤5 years	129 (44.5%)
	6–10 years	112 (38.6%)
	≥11 years	49 (16.9%)

### Measures

The study utilized four validated instruments to assess coaching and athlete-related constructs in Table 2. The Coaching Leadership Style Scale (CLSS; Chelladurai and Saleh, 1980), adapted for Chinese contexts by Yang (2008), measures coaching strategies through 10 items across two subscales (democratic and authoritarian leadership) rated on a 5-point Likert scale (*α* = 0.866 and 0.815, respectively). The Perceived Social Support Scale (PSSS; Zimet et al., 1988), modified with Jiang and Blumenthal revisions, evaluates subjective social support perceptions via 12 items across three domains (family, peer, and significant others) using a 7-point Likert scale (*α* = 0.948). Task and Ego Orientation in Sport Questionnaire (TEOSQ; Duda and Nicholls, 1992), validated for Chinese populations by, captures motivational patterns with 13 items divided into task (7 items) and ego orientation (6 items) subscales on a 5-point Likert scale (*α* = 0.909 and 0.768, respectively). Finally, the Athlete Engagement Questionnaire (AEQ; Lonsdale et al., 2007), cross-culturally validated by Jiang et al. (2023), assesses athlete engagement through 16 items across four dimensions (confidence, vigor, dedication, and enthusiasm) rated on a 5-point Likert scale (*α* = 0.972). All scales demonstrated strong psychometric properties, with internal consistency coefficients exceeding the recommended threshold of *α* > 0.70.

**Table 2:** Elements for measuring coaching leadership style on athlete engagement.

Variable	Item	Content	Reference
CLSS	DL1	1. Seeks athletes’ input on specific competition strategies	Chelladurai and Saleh (1980) and Yang (2008)
	AL1	2. Maintains a cold, distant attitude toward athletes	
	DL2	3. Involves athletes in the decision-making process	
	AL2	4. Dislikes athletes asking questions during instruction	
	DL3	5. Consults athletes on major training and competition matters	
	AL3	6. Demands unconditional acceptance of his/her opinions	
	DL4	7. Encourages athletes to suggest training methods	
	AL4	8. Uses a commanding tone during training and competitions	
	DL5	9. Seeks team input on significant decisions	
	AL5	10. Prohibits others from participating in decision-making	
PSSS	MF1	1. Some people (teachers, classmates, relatives) are present when I encounter problems	Zimet et al. (1988)
	MF2	2. I can share happiness and sadness with others	
	MF3	3. My family provides tangible support to me	
	MF4	4. I receive emotional support from my family when needed	
	MP1	5. Some people (teachers, classmates, relatives) comfort me genuinely	
	MP2	6. My friends genuinely help me	
	MP3	7. I can rely on my friends during difficulties	
	MP4	8. I discuss my problems with my family	
	MS1	9. My friends share happiness and sadness with me	
	MS2	10. There are people (teachers, classmates, relatives) who care about my feelings	
	MS3	11. My family willingly assists me in making decisions	
	MS4	12. I discuss my problems with friends	
TEOSQ	TO1	1. Learning a new technique motivates me to practice more	Duda and Nicholls (1992)
	EO1	2. When I am the only one mastering a technique/skill	
	TO2	3. Learning a technique that makes me feel joyful	
	EO2	4. When I outperform my peers	
	TO3	5. Learning a new technique through effort	
	EO3	6. When others cannot perform as well as me	
	TO4	7. When I train with great effort	
	EO4	8. When others have problems but I do not	
	TO5	9. Learning a technique that motivates me to practice more	
	EO5	10. When I achieve the best/highest performance	
	TO6	11. Mastering a newly learned movement effectively	
	EO6	12. When I am the best performer	
	TO7	13. When I give my maximum effort	
AEQ	C1	1. I believe I can achieve my personal sports goals	Lonsdale et al. (2007) and Jiang et al. (2023)
	V1	2. I am passionate during training and competitions	
	D1	3. I am committed to achieving my sports goals	
	E1	4. I am excited about my sport	
	C1	5. I believe I can succeed in sports	
	V1	6. I feel energetic during training and competitions	
	D1	7. I am determined to achieve my sports goals	
	E1	8. I am enthusiastic about my sport	
	C1	9. I believe I have the skills to succeed in sports	
	V1	10. I feel vigorous during training and competitions	
	D1	11. I fully devote myself to my sport	
	E1	12. I enjoy my sport	
	C1	13. I am confident in my abilities	
	V1	14. I remain highly alert during training and competitions	
	D1	15. I hope to achieve my sports goals through effort	
	E1	16. I find great enjoyment in my sport	

CLSS: DL, democratic leadership; AL, authoritarian leadership. PSSS: F, family; P, peer; S, significant others. TEOSQ: TO, task orientation; EO: ego orientation. AEQ: C, confidence; V, vigor; D, dedication; E, enthusiasm.

### Control variables

In addition to the primary research variables, this study controlled for variables of athletes’ demographic characteristics including gender, age, and years of training to explore the unique effects of coaching leadership style on athlete engagement. The impact of coaching leadership style on athlete engagement may vary based on these demographic factors, and it has been shown that gender and age moderate the impact of leadership style on athlete motivation and engagement (Smith and Smoll, 1990; Vella et al., 2013). In addition, training experience may also influence athletes’ responses to different leadership styles, with more experienced athletes potentially differing in their motivation and engagement patterns from those with less training experience (Horn, 2008). Therefore, controlling for gender, age, and years of training can more clearly analyze the independent effects of coaching leadership style on athlete engagement.

### Data analysis

Descriptive statistics and Pearson’s correlation analysis were implemented using SPSS 26.0 to depict data profiles and explore correlations between variables. Variance inflation factor (VIF) covariance test was conducted, setting VIF > 10 as the criterion for seriousness of multicollinearity. Chain mediation effect analysis was performed using Hayes (2012) model 6, and the bias-corrected percentile Bootstrap method was used to verify the significance of the mediation effect, with the 95% confidence interval not containing 0 as the basis for determining statistical significance (Erceg-Hurn and Mirosevich, 2008). Prior to data analysis, the Harman one-way test was implemented to assess and reduce the effect of common method variance (Podsakoff et al., 2003).

## Results

### Common method bias test

Due to the use of an anonymous survey where all variables were self-reported by the athletes, the findings may have been influenced by common method bias. To address this issue, a Harman one-way test was conducted on all items. The results showed that nine factors had eigenvalues greater than 1, with the largest factor explaining 37% of the variance, which was below the critical criterion of 40%. It indicates that there is no serious common method bias in this study.

### Descriptive statistics and correlation analysis of each variable

Table 3 presents the means, standard deviations, and Pearson correlation coefficients for the studied variables, providing insights into the relationships between coaching leadership styles, perceived social support, task orientation, ego orientation, and athlete engagement. The results indicate a significant positive correlation between democratic leadership style and perceived social support [*r* = 0.428, *p* < 0.001], task orientation [*r* = 0.387, *p* < 0.001], and athlete engagement [*r* = 0.531, *p* < 0.001]. However, no significant relationship was found between democratic leadership style and ego orientation [*r* = 0.007, *p* > 0.05] or autocratic leadership style[*r* = −0.016, *p* > 0.05].

**Table 3:** The correlation of the main study variables (*N* = 290).

	Mean	SD	DLS	ALS	PSS	TO	EO	AE
DLS	3.869	0.919	1					
ALS	2.091	0.990	−0.016	1				
PSS	65.53	13.885	0.428^***^	−0.024	1			
TO	4.128	0.720	0.387^***^	−0.092	0.553^***^	1		
EO	3.421	0.834	0.007	0.223^***^	0.265^***^	0.479^***^	1	
AE	16.562	3.254	0.531^***^	−0.114	0.570^***^	0.625^***^	0.263^***^	1

*p < 0.05; **p < 0.01;***p < 0.001.

Autocratic leadership style, in contrast, showed a significant positive correlation with ego orientation [*r* = 0.223, *p* < 0.001] but no significant relationships with perceived social support [*r* = −0.024, *p* > 0.05], task orientation [*r* = −0.092, *p* > 0.05], or athlete engagement [*r* = −0.114, *p* > 0.05]. This divergence highlights the distinct effects of leadership styles on athletes’ psychological and behavioral outcomes, emphasizing the more constructive role of democratic leadership in fostering positive athlete engagement and motivation.

Further, perceived social support exhibited strong positive correlations with task orientation [*r* = 0.553, *p* < 0.001] and athlete engagement [*r* = 0.570, *p* < 0.001], underscoring its pivotal role as a mediator. Similarly, task orientation demonstrated the highest correlation with athlete engagement [*r* = 0.625, *p* < 0.001], suggesting its critical influence on athletes’ sustained involvement in sports. Ego orientation was positively correlated with task orientation [*r* = 0.479, *p* < 0.001] and athlete engagement [*r* = 0.263, *p* < 0.001], albeit to a lesser extent, reflecting its more limited impact compared to task orientation.

These findings validate several hypotheses (H1a, H2a, H3a, H4a) while rendering others invalid (H1b, H2b, H3b, H4b). The results reinforce the importance of democratic leadership and task orientation in enhancing athlete engagement and the moderating influence of perceived social support.

### Relationship between coaches’ leadership styles and athlete engagement: a chain-mediation model

Based on the correlation analysis between the variables, it was found that the correlation coefficients between coaches’ authoritarian leadership styles and athlete engagement were not significant (*P* > 0.05), and the correlations of perceived social support and athletes’ task orientation were not significant (*P* > 0.05); therefore, only the chain mediating effects of perceived social support and athletes’ task orientation on the relationship between coaches’ democratic leadership styles and athlete engagement were examined. Chain mediating effect was tested. Prior to testing the effects, the predictor variables in the equations were standardized and covariance diagnostics were performed. The results showed that the variance inflation factors for all predictor variables (1.375, 1.989, and 1.568) were less than 5. Therefore, the data used in this study did not suffer from serious covariance problems and were suitable for further testing of the mediating effect. The process plug-in developed by Hayes was used to assess the 95% confidence interval (CI) of the mediating effect of perceived social support and task orientation in the effect of democratic leadership style on athlete engagement (bootstrap sample size of 5,000), and the results of the chain mediation model were developed as shown in Table 4.

**Table 4:** Testing for the multiple mediating effects.

Regression equation (*N* = 290)		Fitting index			Coefficient and significance	
Outcome Variables	Predictor Variables	R	R^2^	F	*β*	t
PSS	Constants	0.428	0.183	64.617	10.123^***^	12.650
	DLS				1.618^***^	8.039
TO	Constants	0.578	0.333	71.689	1.960^***^	10.456
	DLS				0.145^***^	3.455
	PSS				0.099^***^	8.882
AE	Constants	0.725	0.525	105.427	1.882^***^	2.236
	DLS				0.994^***^	6.095
	PSS				0.221^***^	4.639
	TO				1.746^***^	7.743

*p < 0.05; **p < 0.01;***p < 0.001.

**Figure 2:**
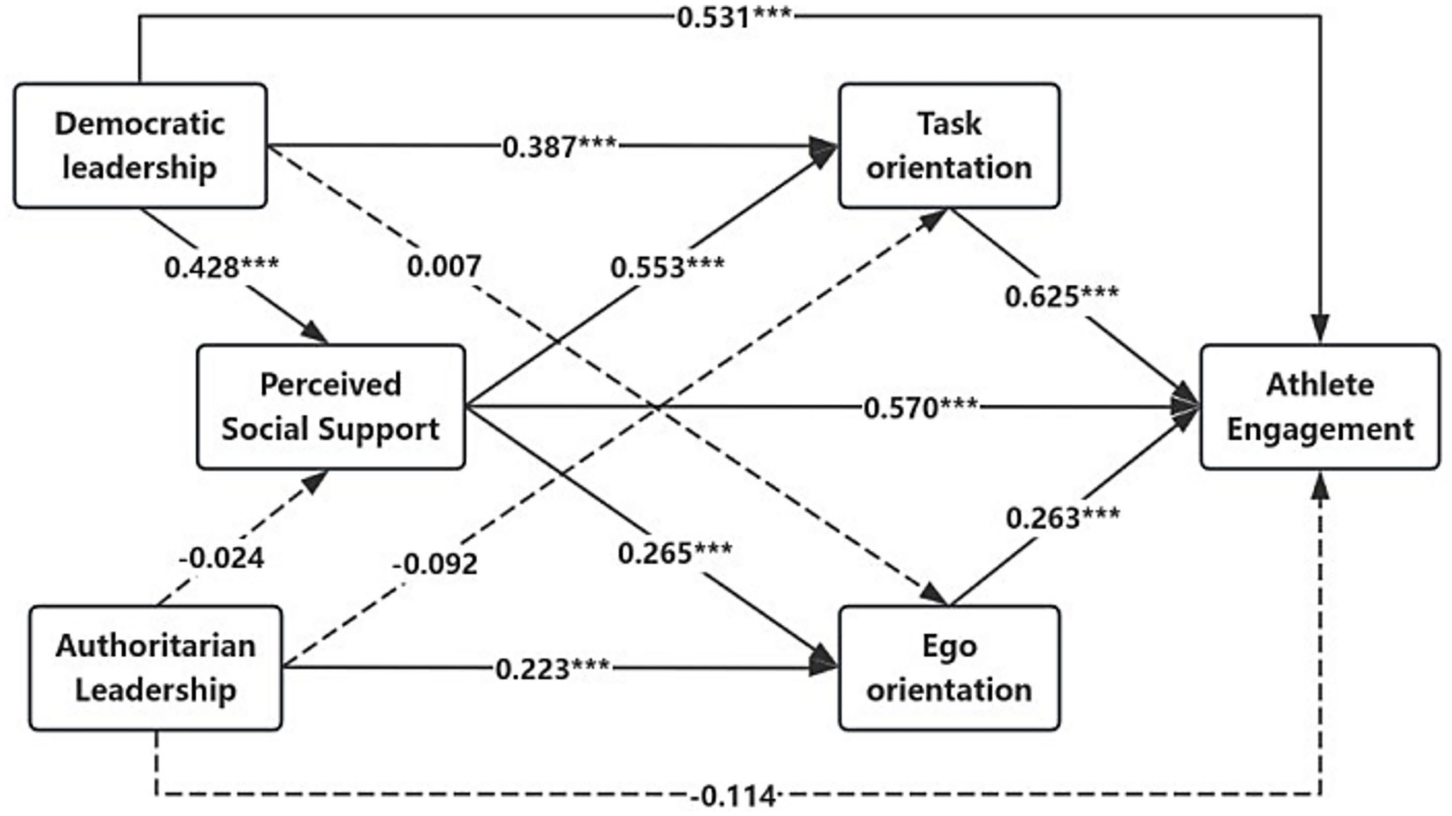
The multiple mediating effects of perceived social support and achievement goal orientation. The dotted line indicates that the path coefficient is not significant.

The results showed that coaches’ democratic leadership style had a positive and significant predictive effect on athlete engagement [*β* = 0.531, *p* < 0.001]. Also, coaches’ democratic leadership styles had a positive and significant predictive effect [*β* = 0.428, *p* < 0.001; *β* = 0.387, *p* < 0.001] on perceived social support and athletes’ task orientation, and hypothesis H1a was supported. Perceived social support had a significant positive predictive effect on athlete task orientation and athlete engagement [*β* = 0.553, *p* < 0.001; *β* = 0.570, *p* < 0.001]. Athlete task orientation was a significant positive predictor of athlete engagement [*β* = 0.625, *p* < 0.001] (see Figure 2).

Table 5 further examination of the mediating effects revealed that the Bootstrap 95% confidence interval for the total indirect effect of perceived social support and athlete task orientation in the effect of democratic leadership styles on athletic commitment was (0.562, 1.251), which did not contain zero, indicating that perceived social support and athlete task orientation play a mediating role. The total indirect effect of the two was 0.888, accounting for 47.18% of the total effect. This mediating effect consisted of three main pathways.

1. Democratic leadership style→perceived social support→athlete engagement [95%CI = (0.172, 0.611), standard error (SE) = 0.115], with a mediating effect of 0.358, accounting for 19.02% of the total effect. Hypothesis H2a was supported.
2. Democratic leadership style→Task orientation→Athlete engagement [95%CI = (0.060, 0.490), standard error (SE) = 0.109], with a mediating effect of 0.252, accounting for 13.39% of the total effect. Hypothesis H3a was supported.
3. Democratic leadership style→Perceived social support→Task orientation→Athlete engagement [95%CI = (0.145, 0.451), standard error (SE) = 0.079, with a mediating effect of 0.079], with a mediating effect of 0.278, accounting for 14.77% of the total effect. Hypothesis H4a was supported.

**Table 5:** The estimates of total, direct and indirect effects of the model.

	Effect	Boot SE	Boot LL CI	Boot UL CI	Relative mediation effect
Total Indirect effect	0.888	0.178	0.562	1.251	47.18%
Indirect effect 1	0.358	0.115	0.172	0.611	19.02%
Indirect effect 2	0.252	0.109	0.060	0.490	13.39%
Indirect effect 3	0.278	0.079	0.145	0.451	14.77%

Indirect effect 1: democratic leadership style→perceived social support→athlete engagement; Indirect effect 2: democratic leadership style→task orientation→Athlete engagement; Indirect effect 3: democratic leadership style→perceived social support→task orientation→athlete engagement.

## Discussion

After controlling for gender, age, years of training, and type of sport, this study found that democratic coaching leadership style had a significant positive effect on athlete participation. This result is consistent with previous research suggesting that democratic leadership styles can significantly enhance intrinsic motivation and participation by satisfying athletes’ feelings of autonomy, competence, and belonging (Deci and Ryan, 1975; Smith and Smoll, 1990; Vella et al., 2024) Self-determination theory suggests that environments that support autonomy help to increase motivation and decrease feelings of disengagement, thereby sustaining athletes’ long-term engagement (Ryan and Deci, 2000; Standage et al., 2003).

In the present study, perceived social support and achievement goal orientation served as mediators, further illustrating how coaching support promotes athletes’ focus on task orientation and self-improvement rather than simply seeking external recognition (Reinboth et al., 2004). These findings suggest that emotional support from coaches not only enhances athletes’ sense of psychological security, but also encourages them to set meaningful goals and stay persistent in the face of challenges, which in turn reduces the risk of burnout (Mageau and Vallerand, 2003).

In addition, this study highlights the importance of democratic leadership in both team and individual sport programs (Curran et al., 2015), where supportive coaching helps athletes to better cope with competitive pressures and increase satisfaction with sport participation, which in turn reduces dropout rates (Weiss and Ferrer-Caja, 2002; Vallerand and Losier, 1999). These findings support existing hypotheses and are consistent with the established sport psychology literature on the effects of leadership style on athlete behavior.

### The direct effect of coach’s leadership style and athletic engagement

The results of the present study found that democratic leadership styles positively predicted athlete engagement, i.e., the more democratic the coach’s leadership style, the more focused the athlete engagement. This is consistent with existing findings (Gunnarsson and Þórisson, 2017; Calvo and Topa, 2019). Furthermore, research has shown that authoritarian leadership styles, on the other hand, have a negative effect on athlete engagement, meaning that the more coaches tend to use controlling or high-pressure management styles (Nasiruddin et al., 2020), the less engaged athletes are (Olsson et al., 2022; Tait et al., 2020). This result is consistent with previous research and emphasizes the positive role that democratic leadership styles play in sports teams. By giving athletes more autonomy and participation, democratic leadership styles not only increase their level of engagement, but also enhance team cohesion and satisfaction (Yasuda, 2019). McMorris and Hale (2006) argued that authoritarian leadership styles may improve discipline and execution in the short term, but in the long term may lead to athlete burnout and an increased psychological stress(Graña et al., 2021), which in turn affects their athletic performance. The non-significant relationship between authoritarian leadership and athlete engagement may reflect cultural nuances in China’s sports environment. Authoritarian coaching, characterized by centralized decision-making, is traditionally prevalent in Chinese sports training systems (Hou, 2024). Athletes in this context may normalize strict control as a standard practice, thereby attenuating its negative association with engagement. Future studies should explore contextual moderators to clarify this phenomenon. Such as dynamics and sport type. These findings further support an athlete-centered coaching model that promotes intrinsic motivation and passion for sport through open communication and supportive approaches (Knight, 2014).

According to Self-Determination Theory, individuals are more intrinsically motivated when they fulfill autonomy, relatedness, and competence. This study suggests that a democratic leadership style can satisfy athletes’ need for autonomy and thus intrinsic motivation by empowering them to be more involved. Therefore, coaches can encourage athletes to participate in the training and decision-making process by respecting their opinions and making them feel valued and recognized in the team. This not only helps to increase athletes’ commitment to their sport, but also enhances their performance in competition and long-term development potential. To foster athlete engagement, sports organizations should prioritize democratic leadership training programs that emphasize collaborative decision-making, open communication, and autonomy support. For instance, coaches could implement weekly team meetings for goal-setting and integrate athlete feedback into training plans. Policymakers might establish mentorship systems pairing experienced democratic coaches with novices to disseminate effective practices. Additionally, institutional support structure should reinforce social support networks to amplify the benefits of democratic leadership.

### The mediating role of perceived social support

This study demonstrated that perceived social support partially influenced the relationship between democratic leadership styles and athlete engagement. That is, the more democratic the coach’s leadership style, the higher the athlete’s level of perceived social support and therefore the higher the state of sport engagement. Furthermore, it was found that the mediating role of perceived social support(Gabana et al., 2017) between democratic leadership styles and athlete engagement implies that coaches, by creating supportive and encouraging environments, can enhance athletes’ sense of belonging and being recognized, which further motivates their sport engagement (Gayles et al., 2018). This finding reveals the critical role of social support in enhancing athletes’ motivation and persistence. Specifically, democratic leadership styles encourage positive interactions among athletes and between athletes and coaches, making them more willing to take on challenges and feel safe and valued on the team (Fogaca, 2021). In this atmosphere of high social support, athletes are more inclined to put their own efforts into training and competition because they feel that they are not only playing for themselves, but also for the trust and expectations of their team and coach (Lee, 2019). These results suggest that coaches should emphasize the establishment of a supportive team culture when adopting a democratic leadership approach to further enhance athletes’ psychological security and engagement levels (Norris et al., 2017). This approach not only helps to enhance athletic performance, but also reduces athletes’ anxiety and stress levels, thereby creating better training and competition outcomes.

Based on Social Support Theory (House et al., 1988), social support plays an important role in individuals’ mental health and stress management. This study reveals the key moderating role of perceived l social support between democratic leadership style and athletes’ sport commitment (Gao et al., 2021). Therefore, coaches should actively build supportive relationships to help athletes feel cared for and supported on the team by providing emotional support, recognition, and encouragement. Such a supportive atmosphere not only reduces athletes’ anxiety, but also enhances their mental toughness, thereby increasing the depth and durability of their athletic engagement.

### The mediating role of sense of achievement goal orientation

The results of this study propose that task orientation partially influences the relationship between democratic leadership styles and athlete engagement. That is, the more democratic the coach’s leadership style, the higher the self-efficacy and positive psychological state the athletes will exhibit, and therefore the higher their state of sport engagement. Task orientation has a significant impact on athletes’ physical and mental health. Further, the mediating role of task orientation suggests that a democratic leadership style not only directly affects athlete engagement, but also indirectly contributes to their engagement level by enhancing their self-efficacy and achievement motivation (Alesi et al., 2019). When coaches adopt a democratic leadership style, athletes feel recognized for their efforts and accomplishments, which in turn enhances their self-efficacy and adherence to goals (Barr et al., 2015). This task-oriented environment allows athletes to focus more on personal growth and skill enhancement rather than solely on winning or losing competitions, which reduces external pressures and enhances long-term commitment and interest in the sport (Lange-Smith et al., 2024). By supporting athletes’ personal goals and developmental pathways, democratic leadership styles help foster intrinsic motivation to maintain a positive mental state and high level of engagement in the face of challenges. Also, task orientation creates a culture within the team that values individual progress and motivates athletes to give their best in the context of psychological well-being (Domingues et al., 2024). In summary, this mediating mechanism further emphasizes the importance of a democratic leadership style combined with task-oriented strategies to help achieve more efficient and sustainable athlete development outcomes.

Achievement Goal Theory (Nicholls, 1984) emphasizes that task orientation can help individuals seek self-development and skill enhancement in the process. The present study suggests that task orientation plays an important role in the chain of pathways through which democratic leadership styles influence athletic engagement. Coaches should emphasize growth and skill-enhancement goals rather than singularly pursuing performance in their daily training so that athletes focus on personal progress and ability development. This not only enhances athletes’ self-efficacy, but also enables them to be more focused and engaged in training and competition.

### The multiple mediating effects

This study found that perceived social support and task orientation are closely related, and they constitute a chain mediating effect that influences the path of democratic leadership style → perceived social support → task orientation → athlete engagement. That is, the more democratic the coach’s leadership style, the higher the level of perceived social support of the athlete, and therefore better task orientation and higher state of athlete engagement. Perceived social support and task orientation are closely related. Higher levels of perceived social support imply that individuals achieve a more positive state in task orienting. Conversely, lower levels of perceived social support represent individuals in a more negative emotional experience in task orienting. This chain-mediated effect reveals how a democratic leadership approach ultimately promotes higher levels of sport engagement by progressively enhancing athletes’ psychological support and task orientation. Specifically, when coaches adopt a democratic leadership style, athletes perceive higher levels of social support, which not only enhances their sense of psychological safety, but also boosts positive attitudes toward training and competition (Jawoosh et al., 2022). In the process, perceived social support provides athletes with emotional support and a sense of belonging, which in turn improves their task orientation to focus more on self-growth and skill development in training rather than being purely outcome-oriented (Pope and Wilson, 2012; De Backer et al., 2021). Higher levels of social support motivate athletes in task orientation, leading to greater self-confidence and positive responses to challenges, resulting in deeper engagement in sport (Jiang et al., 2023). Conversely, lower social support tends to cause athletes to experience negative emotions such as anxiety and stress during task orienting, which in turn inhibits their state of sport engagement (Roby, 2023). Furthermore, this chain-mediated mechanism suggests that task orienting serves as a bridge between social support and athletic engagement, and is able to transform the psychological resources from social support into motivation and sustained engagement for athletes, creating positive feedback (Ulvick, 2015). Coaches should consider the synergistic effects of social support and task orientation when enhancing athletic performance to promote overall psychological well-being and long-term development of athletes (Gray, 2022).

The study demonstrated the chain-mediated effects of perceived social support and task orientation, suggesting that coaches can integrate the two to enhance athletes’ psychological well-being and sport engagement. This integration strategy is consistent with Bandura (1977) theory of self-efficacy, which suggests that by providing positive social support and task orientation, athletes’ self-efficacy can be enhanced (Sullivan and Kent, 2003), resulting in greater self-confidence in the face of challenges. Coaches should provide encouraging feedback and reasonably challenging tasks to progressively enhance athletes’ skills and mental toughness, ultimately leading to sustained high engagement and performance.

### Limitations and implications

This study is not without its limitations. The use of cross-sectional data constrains the ability to establish causal relationships between variables. Additionally, the reliance on convenience sampling introduces potential selection bias, which may affect the generalizability of the findings. Moreover, due to time constraints, the study did not incorporate an intervention component within the questionnaire design. To address these limitations, Future intervention studies could implement short, coach-led workshops that emphasize democratic leadership behaviors to empirically test the chain-mediated pathway identified here. Incorporating an intervention component and conducting follow-up studies would provide more robust evidence to validate the causal relationships among the key variables identified in this research.

## Conclusion

This study examined how democratic leadership styles promote athlete engagement through perceived social support and task orientation, offering novel insights into the psychological and behavioral impact of leadership behaviors. The findings revealed that democratic leadership not only directly enhances athlete engagement but also establishes a clear indirect pathway through perceived social support and task orientation. Notably, task orientation emerged as a critical mediating variable, translating social support into higher intrinsic motivation and engagement by fostering athletes’ focus and effort toward specific goals. This provides a nuanced perspective on the mechanisms linking leadership styles to athlete performance.

The mediating role of task orientation further underscores that perceived social support extends beyond emotional and psychological benefits, functioning as a psychological mechanism that strengthens athletes’ self-efficacy and goal focus. By enhancing task orientation, social support promotes positive psychological states and behaviors, ensuring sustained athletic engagement and performance over time.

These findings hold significant practical implications for sports leadership. Coaches are encouraged to adopt democratic leadership practices that elevate athletes’ perceptions of social support while simultaneously fostering task-oriented behaviors. This dual approach could further unlock athletes’ potential for enhanced engagement and performance. Future research should explore the longitudinal effects of task orientation and consider moderating factors such as cultural and contextual influences to construct a more comprehensive theoretical framework (Woods et al., 2020). By elucidating the mediating role of task orientation, this study contributes to a deeper understanding of how democratic leadership fosters athlete engagement, advancing theoretical and empirical knowledge in sports psychology.

## Data Availability

The datasets presented in this study can be found in online repositories. The names of the repository/repositories and accession number(s) can be found in the article/Supplementary material.
